# Variability of outcome reporting in Hirschsprung’s Disease and gastroschisis: a systematic review

**DOI:** 10.1038/srep38969

**Published:** 2016-12-12

**Authors:** Benjamin Saul Raywood Allin, Amy Irvine, Nicholas Patni, Marian Knight

**Affiliations:** 1 National Perinatal Epidemiology Unit, Oxford, OX37LF, UK; 2 Department of Paediatric Surgery, Oxford Children’s Hospital, Oxford, OX39DU, UK; 3 University of Oxford Medical School Medical Sciences Divisional Office University of Oxford Level 3, John Radcliffe Hospital Oxford OX3 9DU, UK.

## Abstract

Heterogeneity in outcome reporting limits identification of gold-standard treatments for Hirschsprung’s Disease(HD) and gastroschisis. This review aimed to identify which outcomes are currently investigated in HD and gastroschisis research so as to counter this heterogeneity through informing development of a core outcome set(COS). Two systematic reviews were conducted. Studies were eligible for inclusion if they compared surgical interventions for primary treatment of HD in review one, and gastroschisis in review two. Studies available only as abstracts were excluded from analysis of reporting transparency. Thirty-five HD studies were eligible for inclusion in the review, and 74 unique outcomes were investigated. The most commonly investigated was faecal incontinence (32 studies, 91%). Seven of the 28 assessed studies (25%) met all criteria for transparent outcome reporting. Thirty gastroschisis studies were eligible for inclusion in the review, and 62 unique outcomes were investigated. The most commonly investigated was length of stay (24 studies, 80%). None of the assessed studies met all criteria for transparent outcome reporting. This review demonstrates that heterogeneity in outcome reporting and a significant risk of reporting bias exist in HD and gastroschisis research. Development of a COS could counter these problems, and the outcome lists developed from this review could be used in that process.

Systematic reviews comparing key treatments for both Hirschsprung’s Disease and gastroschisis have failed to identify gold standard treatments for either condition^1^,^2^,^3^,^4^. This is likely to be due to a combination of reasons including the small size and retrospective nature of many of the primary studies, but also inadequate data reporting, heterogeneity in outcome definition, and use of surrogate markers of success instead of clinically relevant outcomes^1^. In a recent study reviewing 283 randomly selected Cochrane systematic reviews covering a range of conditions and interventions, Kirkham *et al*. demonstrated that over 30% of the randomised controlled trials included in these systematic reviews either did not publish at all, or did not fully publish the results for their identified primary outcomes^5^. This suggests that the potential for reporting bias is widespread within the medical literature.

Potential reporting bias and potential lack of patient relevance, combined with the fact that chance and confounding are likely to be impacting the results of many Hirschsprung’s Disease and gastroschisis studies^6^ limits the ability of the existing evidence base to help guide clinical management. It has been proposed that the development of core outcome sets for key conditions, combined with increased collaboration can help address these problems^1^,^7^,^8^.

Core outcome sets are groups of outcomes that have been identified through a systematic review and Delphi process, and ratified by key stakeholders as the outcomes that should at a minimum be reported in every study of that condition^8^. They are a tool that has been championed by the COMET (Core Outcome Measures in Effectiveness Trials) initiative as a method for reducing reporting bias, increasing patient relevance of research, and improving meta-analysis. Their use in other conditions has been shown to significantly improve the quality of research being conducted^9^, thereby enabling more evidence-based clinical practice.

Prior to developing a core outcome set it is essential to know which outcomes are currently reported in the published literature, and the quality of their reporting. The aims of this work were therefore to conduct two systematic reviews, in order to identify which outcomes are currently reported in studies comparing surgical interventions for Hirschsprung’s Disease and gastroschisis, determine the completeness of data reporting, and make an empirical assessment of the likelihood of reporting bias in included studies.

## Methods

Two systematic reviews were conducted according to pre-specified protocols that were prospectively registered on PROSPERO (CRD42015024996 and CRD42015025026). Multiple search strategies were used to identify relevant articles from Medline and EMBASE. Search terms were identified from database thesauri and free text relating to either Hirschsprung’s Disease and operative interventions for Hirschsprung’s Disease, or gastroschisis and interventions for gastroschisis, and combined using Boolean operators. Searches were limited to papers published in or after 2010 in order to ensure that identified outcomes were contemporaneous. Full search strategies are provided in supplementary files one and two.

### Study selection and data extraction

Identified titles were assessed for inclusion by three investigators acting independently (BA and AI for Hirschsprung’s Disease, and BA and NP for gastroschisis). Any conflicts were resolved by discussion, with recourse to a fourth investigator (MK) where necessary. Data from included articles were extracted independently for each review by the same two investigators, with conflicts resolved in the same manner. The following data were extracted from all studies: study design, year of study, interventions investigated, population investigated, size of study population, inclusion and exclusion criteria, outcomes reported, whether outcomes were primary or secondary, time-points at which outcomes were measured, which of the criteria for complete reporting the study met, and the ORBIT grade^5^ for each investigated outcome. ORBIT grades range from A-I and are used to denote the completeness of reporting for a particular outcome.

### Inclusion and exclusion criteria

All study designs except case studies and expert opinion were considered for inclusion in the review. Studies where only the abstract was available (e.g. conference proceedings) were not included in the assessment of completeness of data reporting, as this could only be analysed from the full study report. The search was limited to papers published after 2010 in order to ensure that the outcomes identified by the review remained contemporaneous. Only studies including more than 10 infants were eligible for inclusion.

Additionally, Hirschsprung’s Disease studies were eligible for inclusion if they:

1. Compared two or more interventions for infants with biopsy confirmed Hirschsprung’s Disease and
2. Reported outcomes following the primary definitive procedure.

Additionally, Hirschsprung’s Disease studies were excluded if:

1. They only reported outcomes from one intervention without a comparator.
2. They only reported outcomes for infants undergoing a re-do procedure or
3. They only reported outcomes from the non-definitive procedure e.g. stoma formation in infants planned for a staged procedure.

Additionally, gastroschisis studies were eligible for inclusion if they compared two or more methods of visceral reduction and defect closure in infants with gastroschisis, and were excluded if they only reported outcomes from one intervention without a comparator.

Both searches were undertaken in August 2015.

### Outcome description

The primary aim of this study was to generate a list of all outcomes investigated by eligible studies. The median number of outcomes reported per study was calculated as a secondary outcome measure. Similar outcomes were merged to one common term prior to analysis.

Outcome terms were then assigned to one of the five core areas from the OMERACT filter 2.0. The OMERACT filter 2.0 represents five core areas that should be covered by outcomes in order to ensure a full breadth of reporting^10^. These areas are (1) adverse events, (2) life impact, (3) resource use, (4) pathophysiological manifestations, and (5) death.

### Completeness of reporting

A secondary aim of this review was to determine the completeness of outcome reporting in Hirschsprung’s Disease and gastroschisis studies. Harman *et al*. proposed five core questions that could be used to assess how transparently researchers had identified and reported their choice of outcomes^11^, whilst the ORBIT criteria are used to determine whether it is likely that data is missing from the studies report^5^. The percentage of studies meeting all five of Harman *et al*.’s core criteria for complete, transparent outcome reporting, and the percentage of studies reporting full data, as described by the ORBIT criteria were calculated for all outcomes investigated. Harman *et al*.’s five core criteria are:

1. Is the primary outcome clearly stated?
2. Is the primary outcome clearly defined so that another researcher would be able to reproduce its measurement?
3. Are the secondary outcomes clearly stated?
4. Are the secondary outcomes clearly defined?
5. Do the authors explain the use of the outcomes which they have selected?

### Data Synthesis

The number of outcomes reported in each eligible study and the number of times each outcome was reported were counted, and the medians and interquartile ranges calculated. The number and percentage of studies answering yes to all five core questions proposed by Harman *et al*., and the number and percentage with full reporting according to ORBIT criteria for all investigated outcomes were also calculated.

## Results

### Hirschsprung’s Disease included studies

**Figure 1:**
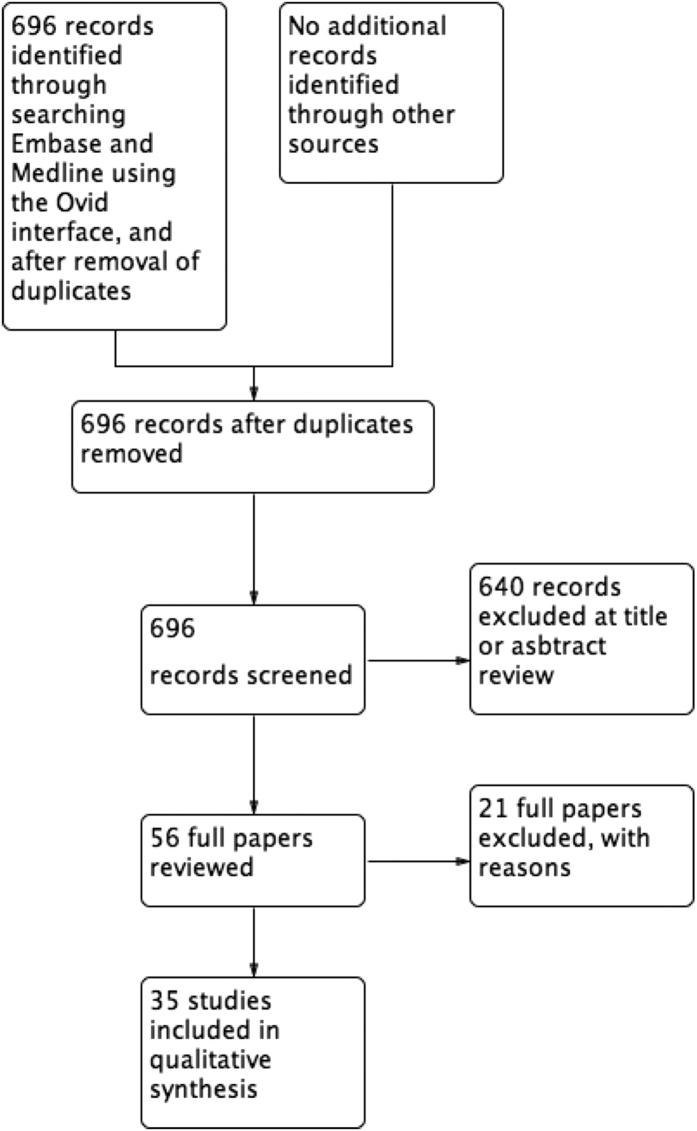
Hirschsprung’s Disease PRISMA flow chart.

The search retrieved 696 unique titles related to Hirschsprung’s Disease, 640 of which were excluded following title and abstract review. Following full paper review, a further 21 papers were excluded with reasons (Supplementary File 3), leaving 35 studies as eligible for inclusion in the review (Fig. 1). Eighteen manuscripts (51%) were retrospective case series, nine (26%) were prospective cohort studies, three (9%) were case control studies, three (9%) were systematic reviews, one (3%) was a registry based study, and one (3%) was a randomised controlled trial. The median number of study participants was 62 (IQR44-156).

### Hirschsprung’s Disease reported outcomes

In the 35 included studies, 95 outcomes were investigated a total of 337 times. Thirty-five outcomes were considered to be too similar to at least one other outcome to be meaningfully differentiated, and these outcomes were therefore mapped to one common term (e.g. continence/incontinence, or frequency of stool/bowel movement frequency). Following this exercise, 74 unique outcomes were identified as having been reported (Table 1).

Outcomes were mapped to the OMERACT filter 2.0. Overall, 33 ‘adverse event’ outcomes, 28 ‘life impact’ outcomes, seven ‘resource use’ outcomes, five ‘pathophysiological manifestation’ outcomes, and one ‘death’ outcome were reported. Adverse event outcomes accounted for 171 of the 338 outcomes that studies investigated (51%), whilst life impact outcomes accounted for 109 (32%).

Overall, faecal incontinence was the most commonly reported outcome and, appearing in 32 studies (91%), the only one to be investigated in more than 75% of studies. Outcomes investigated in more than 50% of studies were enterocolitis (23 studies, 66%), constipation (20 studies, 57%) and length of stay (18 studies, 51%). Thirty-three (45%) of the 74 unique outcomes were only investigated in one study. The median number of outcomes investigated per study was 11 (IQR6-13). Due to the retrospective nature of many of the included studies, and the lack of clearly defined end-points within them, it was not possible to make any meaningful assessment of the ages at which key outcomes were measured.

### Hirschsprung’s Disease Completeness of reporting

Of the 35 included studies, seven (20%) were available only as abstracts and were therefore excluded from assessment of overall quality and completeness of reporting. Of the remaining 28 studies, only 7 (25%) met all five of the core criteria that Harman *et al*. describe for achieving complete, high quality reporting of a study’s results. When assessed against the ORBIT criteria, of the 28 full papers, only seven (25%) fully reported each of the outcomes they set out to investigate (Table 2).

### Gastroschisis included Studies

**Figure 2:**
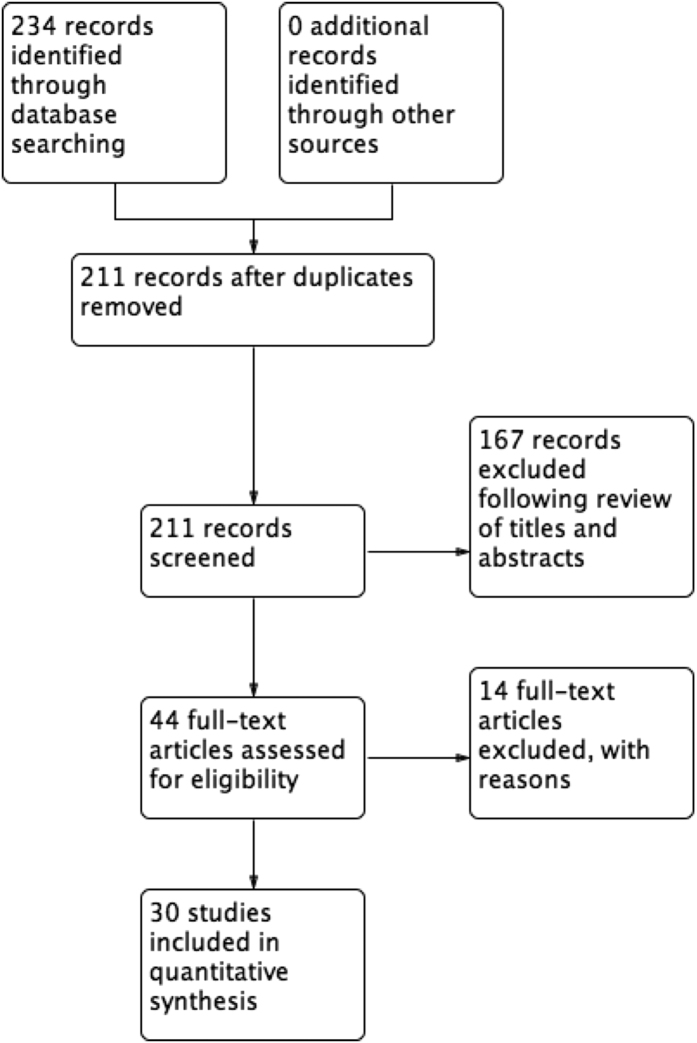
Gastroschisis PRISMA flow chart.

The search strategy returned 234 titles related to gastroschisis, reduced to 211 after exclusion of duplicates. Following review of titles and abstracts, 167 records were excluded. A further fourteen records were then excluded with reasons following full paper analysis (Supplementary File 4), resulting in 30 studies that were deemed eligible for inclusion in the review (Fig. 2). Twenty-two papers (73%) were retrospective case series, four were prospective cohort studies (13%), two were systematic reviews (7%), one was a case-control study (3%), and one was a registry study (3%). There were no eligible randomised controlled trials. The median number of study participants was 122 (IQR 53-285).

### Gastroschisis outcomes

Within the included studies, 102 outcomes were investigated a total of 247 times. Within these 102 outcomes there were 63 that were felt to be too similar to at least one other outcome to be meaningfully differentiated, and these were therefore mapped to one common term. Following this mapping process, there remained 62 unique outcomes (Table 3).

Outcomes were mapped to the OMERACT filter 2.0. Overall, 22 ‘adverse event’ outcomes (35%), 18 ‘pathophysiological manifestation’ outcomes (29%), 12 ‘life impact’ outcomes (20%), nine ‘resource use’ outcomes (15%), and one ‘death’ outcome (1%) were reported. ‘Adverse event’ outcomes were reported 97 times (39% of all reported outcomes), whilst ‘life impact’ outcomes were reported 58 times (23% of all reported outcomes).

Eight of the 62 identified outcomes related to feeding (13%), and five related to mechanical ventilation (8%). These two areas therefore appear to be of interest to researchers. However, the greatest number of studies in which any individual outcome from either of these areas was investigated was 13 (43%), suggesting there is little agreement on what the most important outcome in each area is. Overall, the most commonly investigated outcome was total length of stay, appearing in 24 studies (80%). Mortality (19 studies, 63%), and development of necrotising enterocolitis (16 studies, 53%) were the only other outcomes to be reported in more than 50% of studies. The median number of times an outcome was reported was once (IQR1-2). Thirty-one outcomes (50%) were only reported in one study. The median number of outcomes reported per study was 9 (IQR5-11).

### Gastroschisis completeness of reporting

Of the 30 included studies, three were only available as abstracts, and therefore excluded from analysis of completeness and quality of reporting. Of the remaining 27 studies, none met all five of the core criteria defined by Harman *et al*. for complete, high quality outcome reporting. When assessed against the ORBIT criteria, 12 studies (44%) fully reported each of the outcomes they set out to investigate (Table 4).

## Discussion

This review shows substantial heterogeneity of outcome reporting in Hirschsprung’s Disease and gastroschisis studies. For each condition, over 60 unique outcomes were investigated, only 5% of which were investigated in more than half of the included studies, and over 40% of which were only investigated in one study.

Within Hirschsprung’s Disease research, 32% of investigated outcomes were in the ‘life impact’ core area of the OMERACT filter 2.0, suggesting that there is potential for current research to be investigating outcomes of relevance to patients. The lack of meaningful information on when outcomes are measured however, meant that it was not possible to determine whether these outcomes were investigated at time points where their results would be considered valid (e.g. whether faecal continence was measured in infants who were old enough to be expected to be continent). With 64% of gastroschisis outcomes fitting into either the ‘pathophysiological manifestations’ or ‘adverse events’ core areas, it would suggest that gastroschisis research tends to have a shorter-term focus. With no gastroschisis studies, and only 25% of Hirschsprung’s Disease studies meeting all of the criteria for high quality, complete outcome reporting, and only 25% of Hirschsprung’s Disease and 44% of gastroschisis studies fully reporting all outcome data, there also appears to be a high risk of reporting bias within both research fields.

The search strategy and inclusion criteria for this study were designed to be sensitive whilst maintaining contemporaneity of the identified outcomes. This should provide the most robust basis for development of a core outcome set. Excluding studies conducted prior to 2010 does however introduce the potential for these reviews to miss important outcomes that were fully investigated in high quality studies prior to this point. One example of this is the only randomised controlled trial carried out to date which compares operative primary fascial closure with silo placement for treatment of gastroschisis^12^. Such exclusions have the potential to alter the data on completeness of reporting. However, we were unable to identify any plausible reasons why the study designs used, or quality of reporting should be significantly different pre and post 2010. We therefore do not believe that these exclusions will have significantly altered the conclusions of this review. By including in the current analysis outcomes investigated by systematic reviews, outcomes reported by significant primary studies prior to 2010 (including that of Pastor *et al*.) should also have been captured, and will therefore be included in the list of outcomes used for the development of the core outcome sets.

In order to assess the quality and completeness of outcome reporting, we used tools that were initially designed for assessing randomised controlled trials and systematic reviews. However, it is not unreasonable to expect that any study making active comparison of two interventions meets all five of Harman *et al*.’s core criteria for high quality, transparent reporting, and that they fully report data for all stated a priori outcomes.

It has previously been suggested that variability in outcome definition and reporting is limiting the development of a high quality evidence in Hirschsprung’s Disease and gastroschisis^1^,^2^,^4^. Our review reinforces that message, and adds to it by suggesting that there is also a high risk of reporting bias in research into both conditions, thereby further reducing the reliability of conclusions drawn from the current literature. This high risk of reporting bias echoes what was shown in a large selection of randomised controlled trials included in Cochrane systematic reviews^5^.

The focus of the included studies on pathophysiological manifestations and adverse events, and lack of clarity of time-points for measurement of life impact outcomes raises the possibility that patients and their parents were not involved in determining which outcomes should be investigated by researchers and clinicians. This apparent lack of knowledge as to which outcomes are important to patients and parents in determining the success of gastroschisis and Hirschsprung’s Disease treatment may account in part for the heterogeneity of outcome reporting that our reviews have demonstrated. The difficulties this heterogeneity creates for supporting clinical practice are compounded by the high risk of reporting bias that this review has also demonstrated. Both of these factors suggest that at present, caution should be exercised when using the existing literature to argue in favour of a particular treatment for either condition. There are two concrete steps that can be taken to remedy this situation, firstly, development of core outcome sets for use in gastroschisis and Hirschsprung’s Disease, and secondly, enhanced national and international collaborative research studies and trials. These measures would reduce reporting bias, ease meta-analysis, increase statistical power to answer clinically relevant questions, and improve the patient relevance of research^7^,^8^,^9^.

These systematic reviews provide the evidence that development and implementation of core outcome sets are required for Hirschsprung’s Disease and gastroschisis. The outcomes identified by these reviews could be used as the starting point for a robust Delphi processes involving patients, parents and multi-disciplinary clinical groups to develop such core outcome sets. These will be the first core outcome sets to be developed for any paediatric surgical condition, and we do not wish them to be developed simply as an academic exercise. Hirschsprung’s Disease and gastroschisis were specifically chosen as the first conditions in which to develop paediatric surgical core outcome sets in order to limit the possibility of this occurring. We believe they limit this risk for two reasons. Firstly, both conditions are actively studied at present, and therefore, have a community of researchers who are anticipating the use of the developed core outcome sets. Secondly, there have been contemporaneous, UK-wide cohorts of infants established for both conditions, in whom long-term follow-up studies could be conducted utilising the developed core outcome sets. We have elected to present the results of both systematic reviews as one manuscript, as there are several common themes which lend themselves to being demonstrated best through reporting in a single article. Doing this illustrates to the paediatric surgical community the need for development and implementation of core outcome sets in both of these conditions, and potentially, in others as well.

## Additional Information

**How to cite this article**: Allin, B. S. R. *et al*. Variability of outcome reporting in Hirschsprung’s Disease and gastroschisis: a systematic review. *Sci. Rep.* **6**, 38969; doi: 10.1038/srep38969 (2016).

**Publisher's note:** Springer Nature remains neutral with regard to jurisdictional claims in published maps and institutional affiliations.

## Supplementary Material

Supplementary Tables

## Figures and Tables

**Table 1:** Hirschsprung’s Disease outcomes and number of times reported, grouped according to OMERACT filter 2.0.

Outcome	Number of times reported	Outcome	Number of times reported	Outcome	Number of times reported
**Development-Social**	1	**Medication-laxative**	2	**Narrowing of anastomosis or cuff**	22
**Sleep disturbance**	1	**Dilations**	1	**Impotence**	1
**Depression**	1	**Continence-urinary**	1	**Abdominal distension**	3
**Psychological stress**	1	*Manometry*	1	**Sphincter achalasia**	1
**Cosmetic results**	2	*Bowel Movement*-*first post-op*	3	**Feeding intolerance**	1
**Parenteral nutrition use**	1	*Early pelvic inflammation*	1	**Post-operative infection**	9
**Time until first feed (post-operatively)**	7	*Bladder Dysfunction*	1	**Fever**	1
**Diet tolerated**	1	*Aganglionic bowel remaining*	1	**Abscesses**	5
**Assessment of bowel function**	1	***Death***	7	**Anastomotic Leak**	13
**Bowel Function Score (BFS) questionnaire**	2	**Intra-operative complication**	4	**Pneumonia**	2
**Gastrointestinal Quality of Life Index (GIQLI) questionnaire**	2	**Intra-operative visceral injury**	2	**Dehiscence**	7
**Krickenbeck score**	2	**Colonic torsion**	2	**Enterocolitis**	23
**Sensation of need to defecate**	2	**Ischaemia**	1	**Granuloma**	1
**Voluntary bowel movements**	2	**Conversion**	6	**Colostomy morbidity**	1
**Frequency of bowel movements**	13	**Intra-operative blood loss**	13	**Herniation**	6
**Time to normal bowel habits**	1	**Reoperations**	11	**Colostomy retractions**	1
**Long-term bowel dysfunction**	1	**Excoriation**	10	**Late or adhesional obstruction**	7
**Faecal Impaction**	1	**Anal lacerations**	1	Operation length	17
**Constipation**	20	**Fistula**	1	Time of antibiotic administration	1
**Diarrhoea**	3	**Necrosis of and retraction of colon**	1	Analgesic use post-operatively	2
**Consistency of stool**	3	**Adhesions**	1	ICU admission	1
**Urgency period**	3	**Post-operative ‘complications’**	3	Hospital stay (length)	18
**Requiring nappy**	1	**Early or persistent obstruction**	8	Readmission	2
**Faecal incontinence**	32	**Prolapse**	2	Cost	2
**Encopresis**	1	**Rectal muscularis infection**	1		

**Bold is life impact**, *Italic is pathophysiological manifestations, **Bold italic is mortality***, **underlined bold is adverse events**, and underlined is resource utilisation.

**Table 2:** Number of outcomes reported and completeness of outcome reporting in Hirschsprung’s Disease studies.

Study	Design	Number of infants	Number of outcomes reported	Meets *all* core criteria for complete high quality reporting	Complete data reporting
Ademuyiwa, A. O. *et al*. 2012^13^	Retrospective case series	29	7	No	No
Aworanti, O. M. *et al*.^14^	Retrospective case series	51	4	Yes	Yes
Chen, Y. *et al*.^15^	Systematic Review	131	6	No	Yes
Dahal, G. R. *et al*.^16^	Retrospective case series	11	15	Yes	No
Duncan, N. D. *et al*.^17^	Retrospective case series	45	8	No	No
Dutta, H. K. *et al*.^18^	Prospective cohort	62	13	No	No
El-Sawaf, M. *et al*.^19^	RCT	22	2	Yes	Yes
Fernandez Ibieta, M. *et al*.^20^	Case-Control	38	4	Yes	Yes
Fernandez Ibieta, M. *et al*.^21^	Retrospective case series	220	11	No	No
Gao, M. T. *et al*.^22^	Prospective cohort	70	2	No	No
Giuliani, S. *et al*.^23^	Retrospective case series	29	13	No	No
Gosemann *et al*.^24^	Systematic Review	159	4	No	Yes
Gunnarsdottir, A. *et al*.^25^	Prospective cohort	281	15	No	Yes
Jarvi, K. *et al*.^26^	Case control	101	14	Yes	No
Kim, A. C. *et al*.^27^	Retrospective case series	110	14	No	No
Li, L. Z. *et al*.^28^	Retrospective case series	14	4	No	No
Mabula, J. B. *et al*.^29^	Prospective cohort	181	13	No	No
Miyano, G. *et al*.^30^	Retrospective case series	54	10	No	No
Nah, S. A. *et al*.^31^	Retrospective case series	53	11	No	No
Nasr, A. *et al*.^32^	Case control	52	13	Yes	No
Romero, P. *et al*.^33^	Retrospective case series	11	14	No	No
Stensrud, K. J. *et al*.^34^	Prospective cohort	1555	14	No	No
Stensrud, K. J. *et al*.^35^	Prospective cohort	58	11	No	No
Sulkowski, J. P. *et al*.^36^	registry	218	12	No	No
Tang, S. *et al*.^37^	Retrospective case series	72	10	No	No
Tang, S. T. *et al*.^38^	Retrospective case series	20	11	No	No
Tang, W. *et al*.^39^	Retrospective case series	43	5	No	No
Thomson, D. *et al*.^2^	Systematic Review	50	15	Yes	Yes
Travassos, D. *et al*.^40^	Retrospective case series	90	10	No	No
Van de Ven, T. J. *et al*.^41^	Retrospective case series	153	11	No	No
Visser, R. *et al*.^42^	Retrospective case series	54	14	No	No
Vorobyov, G. I. *et al*.^43^	Retrospective case series	84	7	No	No
Wang, L. *et al*.^44^	Prospective cohort	792	6	No	No
Yang, L. *et al*.^45^	Prospective cohort	1412	10	No	No
Yang, L. *et al*.^46^	Prospective cohort	405	4	No	No

**Table 3:** Gastroschisis outcomes and number of times reported, grouped according to OMERACT filter 2.0.

Outcome	Number of times reported	Outcome	Number of times reported	Outcome	Number of times reported
**Time on*****total*** **parenteral nutrition**	9	*Bacteraemia*	2	**Infection, unspecified or other**	7
**Time on parenteral nutrition**	12	*pH, time acidotic*	1	**Infection, central line related**	6
**Parenteral nutrition ever required**	1	*Kidney dysfunction*	1	**Wound infection or breakdown**	12
**Parenteral nutrition required post-discharge**	1	*Urine output*	3	**Infection with systemic sequelae**	8
**Feeding, initiation of feed in NICU**	1	*Blood pressure, mean arterial*	1	**Infection free survival**	1
**Feeding, full feeds at discharge from NICU**	1	*Need for mesh at closure*	1	**Infection, urinary or respiratory**	3
**Time to first oral feed**	13	*weight* <*10th centile*	1	**Number of transfusions**	1
**Time to full oral feeds**	13	*weight gain*	1	**Silo Complication**	2
**Short Bowel Syndrome**	4	*Hypothyroidism*	1	**Ischaemic bowel**	2
**bowel lengthening procedure required**	1	Length of stay	24	**Anastomotic stricture**	2
**liver transplantation**	1	NICU length of stay	4	**Intestinal perforation**	3
**Neurodevelopmental delay**	1	Discharge, NICU to home	1	**Abdominal compartment syndrome**	7
*Total time on mechanical ventilation*	13	General anaesthesia, number of days, indication	1	**NEC**	16
*Post closure time on mechanical ventilation*	3	Central-line usage ratio (days with central line/hospital days)	1	**Stoma complication**	2
*Ventilation, peak inspiratory pressure*	1	Duration of antibiotics	3	**Adhesional small bowel obstruction**	3
*Ventilation, peak concentration inspired oxygen*	1	Hospital charge	2	**Intestinal Failure Associated Liver Disease**	6
*Post-operative ventilation required*	1	Days to abdominal wall closure	1	**Unplanned reoperation**	9
*Respiratory compromise*	1	rehospitalisation	1	**Reoperation, need for enlargement of gastroschisis defect**	1
*Neonatal Respiratory Distress Syndrome*	1	***Mortality***	19	**Reoperation, need for silo replacement**	1
*Cholestasis*	2	**GI complication**	1	**Umbilical hernia**	3
*Volume of IV fluid required*	1	**Non-GI complication**	1		

**Bold is life impact**, *Italic is pathophysiological manifestations, **Bold italic is mortality***, **underlined bold is adverse events**, and underlined is resource utilisation.

**Table 4:** Number of outcomes reported and completeness of outcome reporting in gastroschisis studies.

Study	Design	Number of infants	Number of outcomes reported	Meets *all* core criteria for complete high quality reporting^11^	Complete data reporting^5^
Alali *et al*.^47^	Retrospective Case Series	86	4	No	Yes
Allin *et al*.^1^	Systematic Review	804	14	No	Yes
Banyard *et al*.^48^	Retrospective Case Series	235	10	No	Yes
Barrett *et al*.^49^	Retrospective Case Series	70	4	No	No
Bradnock *et al*.^50^	Prospective Cohort Study	301	15	No	No
Charlesworth *et al*.^51^	Retrospective Case Series	156	9	No	Yes
Chesley *et al*.^52^	Retrospective Case Series	202	3	No	No
Dariel *et al*.^53^	Retrospective Case Series	64	15	No	Yes
Erdogan *et al*.^54^	Retrospective Case Series	29	12	No	No
Kandasamy *et al*.^55^	Retrospective Case Series	50	13	No	No
Kassa *et al*.^56^	Retrospective Case Series	79	3	No	No
Kunz *et al*.^3^	Systematic Review	1879	8	No	Yes
Lobo *et al*.^57^	Retrospective Case Series	37	10	No	No
Lusk *et al*.^58^	Retrospective Case Series	168	8	No	No
McNamara *et al*.^59^	Retrospective Case Series	30	3	Abstract Only	Abstract Only
Muniz *et al*.^60^	Retrospective Case Series	61	1	Abstract Only	Abstract Only
Murthy *et al*.^61^	Retrospective Case Series	442	11	No	Yes
Niramis *et al*.^62^	Retrospective Case Series	919	4	No	Yes
Orion *et al*.^63^	Retrospective Case Series	80	10	No	No
Owen *et al*.^64^	Prospective Cohort Study	393	9	No	Yes
Payne *et al*.^65^	Case Control Study	116	2	Abstract Only	Abstract Only
Safavi *et al*.^66^	Registry	402	7	No	Yes
Santos Schmidt *et al*.^67^	Retrospective Case Series	45	7	No	No
Schlueter *et al*.^68^	Retrospective Case Series	129	12	No	No
Schmidt *et al*.^69^	Prospective Cohort Study	45	8	No	No
Sirichaipornsak *et al*.^70^	Retrospective Case Series	15	9	No	No
Stanger *et al*. 2010^71^	Retrospective Case Series	679	6	No	Yes
Tsai *et al*.^72^	Retrospective Case Series	44	8	No	No
Van Manen *et al*.^73^	Prospective Cohort Study	167	12	No	Yes
Weil *et al*.^74^	Retrospective Case Series	190	10	No	No
