# Supplementary material for: Variability of outcome reporting in Hirschsprung’s Disease and gastroschisis: a systematic review
Source: Sci Rep. 2016 Dec 12;6:38969. doi: 10.1038/srep38969 (PMC5150519; doi:10.1038/srep38969)
Supplement: Supplementary Tables [file srep38969-s1.doc]

**Supplementary information:**

**Variability of outcome reporting in Hirschsprung’s Disease and gastroschisis: a systematic review**

**Short Title: Outcome reporting heterogeneity in paediatric surgical research**

Benjamin Saul Raywood Allin MBBS, BSc, MRCS ^1,2, *^, Amy Irvine^3#^, Nicholas Patni^3#^, Marian Knight MBChB, MPH, FFPH, DPhil^1^

[benjaminallin@doctors.org.uk](mailto:benjaminallin@doctors.org.uk)

[amy.irvine@jesus.ox.ac.uk](mailto:amy.irvine@jesus.ox.ac.uk)

[nicholas.patni@hertford.ox.ac.uk](mailto:nicholas.patni@hertford.ox.ac.uk)

[marian.knight@npeu.ox.ac.uk](mailto:marian.knight@npeu.ox.ac.uk)

^1^ National Perinatal Epidemiology Unit, Oxford, OX37LF

^2^ Department of Paediatric Surgery, Oxford Children’s Hospital. Oxford, OX39DU

^3^ University of Oxford Medical School, Oxford, OX39DU

**#**These authors contributed equally to this study

*Corresponding author

Correspondence to: BSR Allin, National Perinatal Epidemiology Unit, Richard Doll Building, University of Oxford, old Road Campus, Headington, OX3 7LF [benjaminallin@doctors.org.uk](mailto:benjaminallin@doctors.org.uk), T: 01865 289700 F:01865 289701

**S1 Appendix 1 Hirschsprung’s Disease Search strategies**

*Embase*

1. Hirschsprung Disease.mp. or Hirschsprung disease/
2. recto-sigmoid.mp. [mp=title, abstract, heading word, drug trade name, original title, device manufacturer, drug manufacturer, device trade name, keyword]
3. Hirschsprung*.mp. [mp=title, abstract, heading word, drug trade name, original title, device manufacturer, drug manufacturer, device trade name, keyword]
4. Hirschsprung's Disease.mp. [mp=title, abstract, heading word, drug trade name, original title, device manufacturer, drug manufacturer, device trade name, keyword]
5. Aganglionosis.mp. [mp=title, abstract, heading word, drug trade name, original title, device manufacturer, drug manufacturer, device trade name, keyword]
6. Colon* aganglionosis.mp. [mp=title, abstract, heading word, drug trade name, original title, device manufacturer, drug manufacturer, device trade name, keyword]
7. 1 or 2 or 3 or 4 or 5 or 6
8. laparoscopy/ or laparoscopy.mp.
9. colon resection.mp.
10. pull through.mp. [mp=title, abstract, heading word, drug trade name, original title, device manufacturer, drug manufacturer, device trade name, keyword]
11. pullthrough.mp. [mp=title, abstract, heading word, drug trade name, original title, device manufacturer, drug manufacturer, device trade name, keyword]
12. pull-through.mp. [mp=title, abstract, heading word, drug trade name, original title, device manufacturer, drug manufacturer, device trade name, keyword]
13. laparoscop*.mp. [mp=title, abstract, heading word, drug trade name, original title, device manufacturer, drug manufacturer, device trade name, keyword]
14. open*.mp. [mp=title, abstract, heading word, drug trade name, original title, device manufacturer, drug manufacturer, device trade name, keyword]
15. open approach.mp. [mp=title, abstract, heading word, drug trade name, original title, device manufacturer, drug manufacturer, device trade name, keyword]
16. Duhamel*.mp. [mp=title, abstract, heading word, drug trade name, original title, device manufacturer, drug manufacturer, device trade name, keyword]
17. Soave*.mp. [mp=title, abstract, heading word, drug trade name, original title, device manufacturer, drug manufacturer, device trade name, keyword]
18. Boley*.mp. [mp=title, abstract, heading word, drug trade name, original title, device manufacturer, drug manufacturer, device trade name, keyword]
19. Swenson*.mp. [mp=title, abstract, heading word, drug trade name, original title, device manufacturer, drug manufacturer, device trade name, keyword]
20. (Endo Anal or endoanal or endo-anal).mp. [mp=title, abstract, heading word, drug trade name, original title, device manufacturer, drug manufacturer, device trade name, keyword]
21. (endo rectal or endorectal or endo-rectal).mp. [mp=title, abstract, heading word, drug trade name, original title, device manufacturer, drug manufacturer, device trade name, keyword]
22. (trans abdominal or transabdominal or trans-abdominal).mp. [mp=title, abstract, heading word, drug trade name, original title, device manufacturer, drug manufacturer, device trade name, keyword]
23. (trans anal or transanal or trans-anal).mp. [mp=title, abstract, heading word, drug trade name, original title, device manufacturer, drug manufacturer, device trade name, keyword]
24. biopsy.mp. [mp=title, abstract, heading word, drug trade name, original title, device manufacturer, drug manufacturer, device trade name, keyword]
25. laparoscop* assist*.mp. [mp=title, abstract, heading word, drug trade name, original title, device manufacturer, drug manufacturer, device trade name, keyword]
26. 8 or 9 or 10 or 11 or 12 or 13 or 14 or 15 or 16 or 17 or 18 or 19 or 20 or 21 or 22 or 23 or 24 or 25
27. 7 and 26

*Medline*

1. Hirschsprung Disease.mp. or Hirschsprung disease/
2. recto-sigmoid.mp. [mp=title, abstract, original title, name of substance word, subject heading word, keyword heading word, protocol supplementary concept word, rare disease supplementary concept word, unique identifier]
3. Hirschsprung*.mp. [mp=title, abstract, original title, name of substance word, subject heading word, keyword heading word, protocol supplementary concept word, rare disease supplementary concept word, unique identifier]
4. Hirschsprung's Disease.mp. [mp=title, abstract, original title, name of substance word, subject heading word, keyword heading word, protocol supplementary concept word, rare disease supplementary concept word, unique identifier]
5. Aganglionosis.mp. [mp=title, abstract, original title, name of substance word, subject heading word, keyword heading word, protocol supplementary concept word, rare disease supplementary concept word, unique identifier]
6. Colon* aganglionosis.mp. [mp=title, abstract, original title, name of substance word, subject heading word, keyword heading word, protocol supplementary concept word, rare disease supplementary concept word, unique identifier]
7. 1 or 2 or 3 or 4 or 5 or 6
8. laparoscopy/ or laparoscopy.mp.
9. colon resection.mp.
10. pull through.mp. [mp=title, abstract, original title, name of substance word, subject heading word, keyword heading word, protocol supplementary concept word, rare disease supplementary concept word, unique identifier]
11. pullthrough.mp. [mp=title, abstract, original title, name of substance word, subject heading word, keyword heading word, protocol supplementary concept word, rare disease supplementary concept word, unique identifier]
12. pull-through.mp. [mp=title, abstract, original title, name of substance word, subject heading word, keyword heading word, protocol supplementary concept word, rare disease supplementary concept word, unique identifier]
13. laparoscop*.mp. [mp=title, abstract, original title, name of substance word, subject heading word, keyword heading word, protocol supplementary concept word, rare disease supplementary concept word, unique identifier]
14. open*.mp. [mp=title, abstract, original title, name of substance word, subject heading word, keyword heading word, protocol supplementary concept word, rare disease supplementary concept word, unique identifier]
15. open approach.mp. [mp=title, abstract, original title, name of substance word, subject heading word, keyword heading word, protocol supplementary concept word, rare disease supplementary concept word, unique identifier]
16. Duhamel*.mp. [mp=title, abstract, original title, name of substance word, subject heading word, keyword heading word, protocol supplementary concept word, rare disease supplementary concept word, unique identifier]
17. Soave*.mp. [mp=title, abstract, original title, name of substance word, subject heading word, keyword heading word, protocol supplementary concept word, rare disease supplementary concept word, unique identifier]
18. Boley*.mp. [mp=title, abstract, original title, name of substance word, subject heading word, keyword heading word, protocol supplementary concept word, rare disease supplementary concept word, unique identifier]
19. Swenson*.mp. [mp=title, abstract, original title, name of substance word, subject heading word, keyword heading word, protocol supplementary concept word, rare disease supplementary concept word, unique identifier]
20. (Endo Anal or endoanal or endo-anal).mp. [mp=title, abstract, original title, name of substance word, subject heading word, keyword heading word, protocol supplementary concept word, rare disease supplementary concept word, unique identifier]
21. (endo rectal or endorectal or endo-rectal).mp. [mp=title, abstract, original title, name of substance word, subject heading word, keyword heading word, protocol supplementary concept word, rare disease supplementary concept word, unique identifier]
22. (trans abdominal or transabdominal or trans-abdominal).mp. [mp=title, abstract, original title, name of substance word, subject heading word, keyword heading word, protocol supplementary concept word, rare disease supplementary concept word, unique identifier]
23. (trans anal or transanal or trans-anal).mp. [mp=title, abstract, original title, name of substance word, subject heading word, keyword heading word, protocol supplementary concept word, rare disease supplementary concept word, unique identifier]
24. biopsy.mp. [mp=title, abstract, original title, name of substance word, subject heading word, keyword heading word, protocol supplementary concept word, rare disease supplementary concept word, unique identifier]
25. laparoscop* assist*.mp. [mp=title, abstract, original title, name of substance word, subject heading word, keyword heading word, protocol supplementary concept word, rare disease supplementary concept word, unique identifier]
26. 8 or 9 or 10 or 11 or 12 or 13 or 14 or 15 or 16 or 17 or 18 or 19 or 20 or 21 or 22 or 23 or 24 or 25
27. 7 and 26

**S2 Appendix 2 Gastroschisis Search Strategy**

*Embase*

1. gastroschisis.mp. or gastroschisis/
2. birth defects.mp. or birth defect/
3. abdominal wall defects.mp. or abdominal wall defect/
4. 1 or 2 or 3
5. silo.mp. or *surgical technique/
6. (primary adj3 closure).mp. [mp=title, abstract, heading word, drug trade name, original title, device manufacturer, drug manufacturer, device trade name, keyword]
7. (traditional adj3 closure).mp. [mp=title, abstract, heading word, drug trade name, original title, device manufacturer, drug manufacturer, device trade name, keyword]
8. bianchi.mp.
9. (sutureless adj3 closure).mp. [mp=title, abstract, heading word, drug trade name, original title, device manufacturer, drug manufacturer, device trade name, keyword]
10. 5 or 6 or 7 or 8 or 9
11. 4 and 10

*Medline*

1. gastroschisis.mp. or Gastroschisis/
2. birth defects.mp. or Congenital Abnormalities/
3. abdominal wall defects.mp. or Abdominal Wall/
4. 1 or 2 or 3
5. silo.mp.
6. (primary adj3 closure).mp. [mp=title, abstract, original title, name of substance word, subject heading word, keyword heading word, protocol supplementary concept word, rare disease supplementary concept word, unique identifier]
7. (traditional adj3 closure).mp. [mp=title, abstract, original title, name of substance word, subject heading word, keyword heading word, protocol supplementary concept word, rare disease supplementary concept word, unique identifier]
8. bianchi.mp. or Digestive System Surgical Procedures/
9. (sutureless adj3 closure).mp. [mp=title, abstract, original title, name of substance word, subject heading word, keyword heading word, protocol supplementary concept word, rare disease supplementary concept word, unique identifier]
10. 5 or 6 or 7 or 8 or 9
11. 4 and 10

**S3 Appendix 3 Reasons for exclusion of Hirschsprung’s Disease studies at full paper stage**

| **Author** | **Title** | **Year** | **Reason for Exclusion** |
| --- | --- | --- | --- |
| **Ademuyiwa, AO. Et al** | Swenson’s pull-through in older children and adults: Peculiar peri-operative challenges of surgery | 2011 | No active comparison of outcomes between different surgical techniques |
| **Akilov, K. et al** | Hirschsprung's disease as a cause of chronic constipation in children | 2013 | Abstract only – no evidence of active comparison of outcomes between different surgical techniques |
| **Baxter, KJ. Et al** | Hirschsprung's disease in the preterm infant: Implications for diagnosis and outcome | 2013 | Abstract only – no evidence of active comparison of outcomes between different surgical techniques |
| **Bradnock, TJ. Et al** | Primary laparoscopic-assisted endorectal pull-through for Hirschsprung's disease: 5-year outcome data for a national cohort of 28 cases | 2011 | Abstract only – no evidence of active comparison of outcomes between different surgical techniques |
| **Doodnath, R. Et al** | A systematic review and meta-analysis of Hirschsprung's disease presenting after childhood | 2010 | No active comparison of outcomes between different surgical techniques |
| **Downey, EC. Et al** | Hirschsprung disease in the premature newborn: A population based study and 40-year single center experience | 2015 | No active comparison of outcomes between different surgical techniques |
| **Ekenze, SO. Et al** | Problems and outcome of Hirschsprung's disease presenting after 1 year of age in a developing country | 2011 | No active comparison of outcomes between different surgical techniques |
| **Hotouras, A. Et al** | Stoma related morbidity in infants operated for Hirschsprung's disease: A 17 years single centre experience | 2012 | Abstract only – no evidence of active comparison of outcomes between different surgical techniques |
| **Huang, EY. Et al** | Changes in hospital utilization and management of Hirschsprung disease: Analysis using the kids' inpatient database | 2013 | No active comparison of outcomes between different surgical techniques |
| **Ismail Lotfalla, M. Et al.** | Laparoscopic assisted modified duhamel technique using transanal staplers | 2014 | Abstract only – no evidence of active comparison of outcomes between different surgical techniques |
| **Kapuller, V. Et al** | Transanal approach for Hirschsprung's disease: Three centers experience | 2010 | Abstract only – no evidence of active comparison of outcomes between different surgical techniques |
| **Kholostova, V. V. Et al** | Surgical treatment of Hirschsprung’s disease total form in children | 2014 | Abstract only – no evidence of active comparison of outcomes between different surgical techniques |
| **Ksia, A. Et al** | Soave transanal one-stage endorectal pull-through in the treatment of Hirschsprung's disease of the child above two-year-old: a report of 20 cases | 2013 | No active comparison of outcomes between different surgical techniques |
| **Laughlin, D. M. Et al** | Total colonic aganglionosis: A systematic review and meta-analysis of long-term clinical outcome | 2012 | No active comparison of outcomes between different surgical techniques |
| **Levitt, M. A. Et al** | Transanal, full-thickness, Swenson-like approach for Hirschsprung disease | 2013 | No active comparison of outcomes between different surgical techniques |
| **Nouira, F. Et al** | Surgical management of recto-sigmoid Hirschsprung's disease | 2012 | Abstract only – no evidence of active comparison of outcomes between different surgical techniques |
| **Parsons, C. Et al** | Persistent Faecal Incontinence following surgery for Hirschsprung disease | 2011 | Abstract only – no evidence of active comparison of outcomes between different surgical techniques |
| **Sharma, S. Et al** | Hirschsprung's disease presenting beyond infancy: Surgical options and postoperative outcome | 2012 | No active comparison of outcomes between different surgical techniques |
| **Sowande, O. Et al** | Ten-year experience with the Swenson procedure in Nigerian children with Hirschsprung's disease | 2011 | No active comparison of outcomes between different surgical techniques |
| **Travassos, D. V. Et al** | Is complete resection of the aganglionic bowel in extensive total aganglionosis up to the middle ileum always necessary? | 2011 | No active comparison of outcomes between different surgical techniques |
| **Zhang, J. S. Et al** | Transanal rectal mucosectomy and partial internal anal sphincterectomy for Hirschsprung's disease | 2014 | Abstract only – no evidence of active comparison of outcomes between different surgical techniques |

**S4 Appendix 4 Reasons for exclusion of Gastroschisis studies at full paper stage**

| **Author** | **Title** | **Year** | **Reason for Exclusion** |
| --- | --- | --- | --- |
| **Baumann, S. Et al** | Gastroschisis: A retrospective single center experience | 2012 | Abstract only – no evidence of active comparison of outcomes between different surgical techniques |
| **Chandra, P. Et al.** | Outcomes of surgically treated babies with gastroschisis - A 10-year experience | 2010 | Abstract only – no evidence of active comparison of outcomes between different surgical techniques |
| **Emil, S. Et al** | Contemporary 2-year outcomes of complex gastroschisis | 2010 | No active comparison of outcomes between different surgical techniques |
| **Gupta, V. Et al** | Neurodevelopment outcome at one year of age of infants with gastroschisis | 2014 | Abstract only – no evidence of active comparison of outcomes between different surgical techniques |
| **Kang, R. Et al** | Congenital abdominal wall defects: A six-year experience at Kosair children's hospital | 2012 | Abstract only – no evidence of active comparison of outcomes between different surgical techniques |
| **Lasege, T. B. Et al** | Gastroschisis treatment and outcomes at Kosair children's hospital: A ten-year review | 2010 | Abstract only – no evidence of active comparison of outcomes between different surgical techniques |
| **Manson, J. Et al** | Gastroschisis: A multi-centre comparison of management and outcome | 2012 | Abstract only – unable to determine eligibility therefore excluded |
| **Martinez Criado, Y. Et al** | Modifiable prognostic factors in the morbidity-mortality of gastroschisis | 2012 | No active comparison of outcomes between different surgical techniques |
| **Minutillo, C. Et al** | Growth and developmental outcomes of infants with gastroschisis at one year of age: A retrospective study | 2013 | No active comparison of outcomes between different surgical techniques |
| **Sawicka, E. Et al** | Influence of selected factors on the treatment and prognosis in newborns with gastroschisis on the basis of own experience | 2013 | No active comparison of outcomes between different surgical techniques |
| **Tannuri, A. C. Et al.** | Evolution of critically ill patients with gastroschisis from three tertiary centers | 2011 | No active comparison of outcomes between different surgical techniques |
| **Tarca, E. Et al** | Gastroschisis treatment: What are the causes of high morbidity and mortality rates? | 2013 | No active comparison of outcomes between different surgical techniques |
| **Toro, M. N. H.** | Management of abdominal wall defects (gastroschisis and omphalocele) at Hospital Universitario San Vicente de Paul, in Medellin, Colombia, 1998-2006 | 2010 | Mixed gastroschisis and exomphalos cases, unable to separate |
| **Van Manen, M. Et al.** | Early childhood outcomes of infants born with gastroschisis | 2013 | No active comparison of outcomes between different surgical techniques |
